# Education and HIV incidence among young women in KwaZulu-Natal: An association but no evidence of a causal protective effect

**DOI:** 10.1371/journal.pone.0213056

**Published:** 2019-03-04

**Authors:** Dick Durevall, Annika Lindskog, Gavin George

**Affiliations:** 1 Department of Economics, School of Business, Economics and Law, University of Gothenburg, Sweden; 2 Department of Economics, School of Business, Economics and Law, University of Gothenburg, Sweden; 3 HEARD, University of KwaZulu-Natal, Durban, South Africa; University of Louvain, BELGIUM

## Abstract

We examine the relationship between school attendance and HIV incidence among young women in South Africa. Our aim is to distinguish a causal effect from correlation. Towards this end, we apply three methods to population-based longitudinal data for 2005–2012 in KwaZulu-Natal. After establishing a negative association, we first use a method that assesses the influence of omitted variables. We then estimate models with exclusion restrictions to remove endogeneity bias, and finally we estimate models that control for unobserved factors that remain constant over time. All the three methods have strengths and weaknesses, but none of them suggests a causal effect. Thus, interventions that increase school attendance in KwaZulu-Natal would probably not mechanically reduce HIV risk for young women. Although the impact of school attendance could vary depending on context, unobserved variables are likely to be an important reason for the common finding of a negative association between school attendance and HIV incidence in the literature.

## Introduction

Although HIV infections are decreasing in most countries, about 800 000 individuals in Eastern and Southern Africa became infected in 2016. In South Africa, there were 270 000 new HIV infections, increasing the number of infected to 7.1 million people [1]. Since providing antiretroviral treatment to a rapidly growing number of HIV-positive citizens is a major challenge, the international effort to deal with HIV needs to focus much more strongly on prevention. Currently, keeping girls in school is considered one of the most powerful prevention methods for reducing HIV among adolescent girls across Africa [2, 3, 4]. And large investments have been devoted towards this end. For example, it is a key component of the DREAMS program, a USD 385 million partnership that aims to reduce HIV infections among adolescent girls and young women in South Africa and nine other sub-Saharan African countries during the next few years [5].

Several studies report that education has a protective effect on HIV infection, but most of them focus on correlations [6, 7, 8, 9, 10] rather than casual effects. There exist a few randomized control trials that compare girls who attend school because of some type of intervention with a control group of girls who do not attend school. Still, they all suffer from the inability to separate the effect of the intervention that make girls attend school, such as cash transfers, from the effect of schooling itself [11, 12]. Three studies attempt to estimate a causal effect of schooling on HIV infection using observational data [13, 14, 15]. However, only one study finds an effect on adults’ HIV infection; men and women who completed more years of schooling because of an education reform in Botswana had a lower probability of being HIV positive several years later [15]. The other two studies find that increased enrollment in secondary school reduced risky sexual behavior and improved knowledge, which might have led to a decline in HIV infections [13, 14].

In this paper we focus on the impact of *attending* school on HIV incidence. The previous literature contains studies that analyze the effect of attendance and of school attainment, i.e., on adults having completed a certain number of years of schooling. However, usually no distinction is made between them even though the effect on HIV infection of school attendance probably differs from that of school attainment. Girls in school are in their early twenties or younger and both their behavior and risk environment differ from women in the mid-twenties and older who often are married and have children and who might participate in the labor market. While the short-term impact is only part of the picture, it is an important part. Many girls are infected at a young age, and the timing of infections is likely to matter for the dynamics of the epidemic and the overall impact of interventions [16]. Moreover, interventions are usually expected to affect HIV infections within a year or two.

We use population-based longitudinal data for 2005–2012 from KwaZulu-Natal in South Africa to analyze whether attending school reduces the risk of HIV infection among women aged 15–24. The data include annual HIV incidence so we can determine when infection occurred; almost all other datasets with HIV testing are cross-sectional. We begin by establishing a negative correlation between school attendance and HIV infection, and then investigate whether this correlation appears to be due to an underlying causal protective effect or if it is due to selection, i.e., that unobserved factors determine both school attendance and, through sexual behavior, HIV infection. To do so, we apply empirical strategies often used in econometrics. We first use an approach developed by Altonji et al. [17] to evaluate the sensitivity of the estimated effect of school attendance on HIV incidence to unobserved factors. Thereafter we estimate a bivariate probit model with two exclusion restrictions: the distance to nearest secondary school and the difference in distance between the nearest and second nearest secondary school. If the exclusion restrictions hold, i.e. if the distance variables predict school attendance but not HIV infection in any other way (that we do not control for), this will give us the causal effect of school attendance on HIV infection [18, 19, 20]. We then account for time-constant unobserved effects by estimating the correlated random effects probit model. This will give us the causal effect if unobserved factors that matter for both school attendance and HIV infection, such as family background and time preferences, remain constant over time [20, 21]. Finally, to check for robustness we also do the analysis with linear probability models, applying the approach developed by Oster [22] to evaluate the sensitivity to selection on unobserved factors, and estimating linear probability models with instrumental variables and models that control for time-constant unobserved factors (individual fixed effects) [20]. We do not use logistic regression, the standard approach in epidemiology, since it is not suitable for addressing questions of causality with our data. In particular, it is not straightforward to estimate bivariate logistic models, instead of bivariate probit models, which allow for both causal estimations based on exclusion restrictions and an analysis of sensitivity to selection, i.e. that the variable of interest is endogenous. In any case, logistic and probit regressions typically give very similar results [20].

Our results suggest that unobserved differences across the girls who attend school and those who drop out explain most of the association. Thus, we do not find support for the idea that policies that increase school attendance automatically reduce HIV infections among young women, at least not in the short-run. Given the scarcity of evidence, this paper makes an important contribution to the literature that tries to find the causal impact of attending school on HIV infection. We also contribute to the broader literature on education and risky health behavior [23], as well as to the even larger literature on education and health in general [24].

The following section gives a brief description of earlier studies on schooling and HIV, while Section 3 describes the study area and the data, and Section 4 describes the empirical strategy. Section 5 presents the empirical analysis, and Section 6 provides discussion and conclusions.

## Empirical evidence on the relationship between education and HIV

In this section we review the empirical evidence on the relationship between education and HIV. S1 Appendix describes about the potential links between education and HIV, and the differences between attendance and attainment.

During the early stages of the epidemic, HIV spread faster among the wealthy and well-educated [25, 26]. This was most likely due to the combined effect of differences in lifestyle and a lack of knowledge about HIV. Over time, the pattern of infections has changed in most countries, and several studies show that prevalence nowadays is lower among well-educated adults compared to others [27]. And, as documented by Hardee et al. [10], at least 15 studies find a negative association between education and the risk of HIV. Though these studies control for confounding factors to different degrees, none is likely to completely control for all of them. In fact, most studies use cross-sectional data without attempting to identify a casual effect.

Cross-sectional studies on South African data include Pettifor et al. [28] and Peltzer et al. [29], which find lower infection rates among young women who have completed secondary school than among those who have not, and Hargreaves et al. [30] who find a negative association between school attendance and HIV rates for young men, and between school attendance and sexual risk behaviors for women.

Two studies analyze panel data from sub-Saharan Africa: Bärnighausen et al. [6] and Santelli et al. [9]. Bärnighausen et al. [6] use two rounds of data from a rural area in KwaZulu-Natal, for 2003/2004 and 2005, to determine whether socioeconomic factors affect HIV incidence for women aged 15–49 and men aged 15–54. The main finding is that one extra year of education reduces the risk of acquiring HIV by 7%. Santelli et al. [9] analyze data from sexually experienced Ugandan youth (15–24 years) enrolled in the Rakai Community Cohort Study, 1999–2008. They find that school attendance reduces female HIV infection sharply, with an adjusted Incidence Risk Ratio of 0.22. Although these studies control for reverse causation and a number of observables that are correlated with HIV, they do not control for selection bias caused by unobserved factors such as patience, family background, etc.

As mentioned, three studies employ strategies to identify a causal effect, i.e. they attempt to use variation in schooling which is exogenous and therefore not related to the unobserved factors that could influence sexual behavior directly [13,14,15]. Alsan and Cutler [13] use distance to the nearest secondary school as an instrument. Because their data does not include HIV testing, they focus on the age at sexual debut, a proximate determinant, instead of HIV infection. The main finding is that education delays sexual debut. Using model simulations that link HIV to sexual debut, they suggest that the expansion of girls’ secondary school enrollment in Uganda accounted for between one-sixth and one-half of the decline in HIV prevalence between 1988 and 1995.

Agüero and Bharadwaj [14] use the reform of the educational system in Zimbabwe after independence in 1980 to identify a casual effect; secondary school enrolment of black Zimbabwean students rose sevenfold over the following decade. The study finds that school attainment affects knowledge about HIV and health behavior, but it is unable to detect a significant effect on HIV infection rates.

The third study, De Neve et al. [15], uses a change in the grade structure in Botswana in 1996 that increased educational attainment for cohorts born after 1980. They find that one more year of secondary schooling reduces the accumulated risk of infection by 12 percentage points for women and 5 percentage points for men.

A few randomized controlled trials indirectly test the effect of education on HIV incidence, with mixed results. A cash transfer program in Malawi increased school attendance and reduced HIV infections, but the effects could be due to the cash and/or the increased school attendance [11]. In fact, one study suggests that the effect on HIV might be due to the cash transfers, since it was observed also for unconditional cash transfers, which did not increase schooling [31]. Another study of the effect on HIV acquisition of cash transfers conditional on school attendance among young South African women finds no effect of the transfers on HIV infection or school attendance, but young women who dropped out of school were three times more likely to become HIV infected than the others [28]. However, this finding might be due to unobserved differences between those who dropped out and the average young woman.

Finally, an impact evaluation of girls in Kenya finds that education subsidies reduced pregnancy but not STIs (sexually transmitted infections), while HIV information in school did not reduce STIs or pregnancy, but led to a shift from out-of-wedlock to within marriage pregnancies [32]. However, when a combined program of education subsidies and HIV information was offered, both STIs and pregnancy declined; the number of HIV infections was too small to be used as an outcome variable. The authors set up a theoretical model and show that their results are inconsistent with models where STI infection and pregnancy are the outcomes of a single common determinant: unprotected sex. They propose a model with a distinction between committed relationships, which are likely to end in marriage in the event of pregnancy, and casual relationships, which are not likely to end in marriage. The likelihood of pregnancy is higher in committed relationships while the likelihood of STIs (and presumably HIV) is higher in casual relationships.

To conclude, there is some support for the claim that higher *school attainment* reduces HIV in the long run, but the evidence for an effect of *school attendance* is not strong [31, 33]. Most studies focus on associations, and those that evaluate cash transfer programs do not distinguish between the effect of schooling and the benefits of receiving cash, which, for example, could reduce transactional sex.

## The data

**Fig 1:**
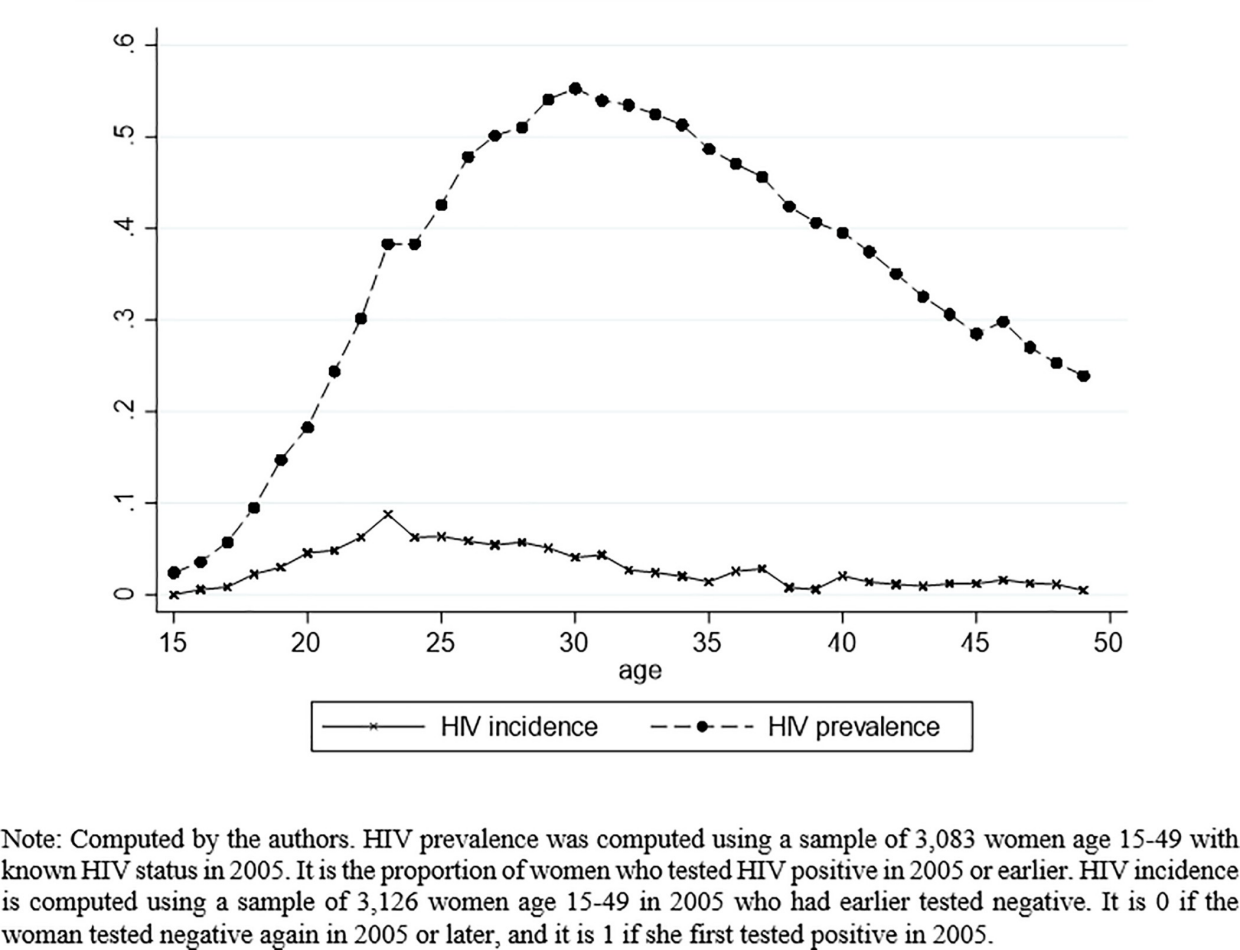
Female HIV prevalence and HIV incidence in 2005 by age.

The data are from a population-based longitudinal surveillance of 85,000 people who are members of approximately 11,000 households [34]. It is conducted by the Africa Health Research Institute in a predominantly rural community in KwaZulu-Natal. It is one of the poorest communities in South Africa. HIV is very common, as can be seen in Fig 1, which shows female HIV prevalence and incidence rates in 2005 by age. The HIV prevalence peaks at over 50%, for women aged 29–30, and the HIV incidence approaches 10% at age 23.

We use data for the period January 2005 to June 2012, i.e. 8 rounds. The data we have access to are fully anonymized.

Blood samples for HIV testing were collected from men and women aged 15 and above. To create an annual indicator of HIV incidence we use information about the first time a woman was tested HIV negative, the last time she was tested negative, and the first time she was tested positive. The indicator is 0 if she tests negative in a year and 1 if she tests positive for the first time in that year. Not everyone took the HIV test every year, so HIV incidence is coded 0 if a woman tested negative after a year with missing data, and it is coded as missing if she tested negative before and positive after a year with missing data. After a woman has tested positive, she is not part of the estimation sample for subsequent years. Hence, our estimation sample consists of young women who were HIV negative the previous time they were tested. We performed robustness checks where HIV incidence was coded as 1 only if the woman tested negative in the year before, or when women with missing years between the last HIV negative test and the first HIV positive test were excluded from the sample.

The sample is also restricted to woman aged 15 to 24 who have not completed secondary education. The share of the young women who have completed secondary school increases with age. At age 19 –when young women should have completed secondary school if they followed their age-appropriate grade throughout– 18.1% have completed secondary school, while 43.8% have completed secondary school by age 24. The reason we restrict the analysis to young women who have not completed secondary school is that we do not want to mix up the effect of not attending secondary school when you should with the effect of either completing secondary school fast or of attending or not attending tertiary education after completing secondary education.

In South Africa, primary school spans from grade 1 to grade 7 and secondary school from grade 8 to grade 12. Schooling is compulsory until age 15 or until grade 9 is completed; therefore, all the young women in the estimation sample could legally leave school if they wish. School attendance is observed in the data each year. Respondents tell if they attend school full-time, part-time or not at all. We measure school attendance with a binary variable indicating reported full-time attendance in the current year. Very few young women report part-time attendance, so the results do not change if school attendance is measured by full- or at least part-time attendance. Girls can, and do, move in and out of school. We do not distinguish between those who are out of school temporarily and those who have dropped out completely. In the estimations sample, 23,92% of young women who did not attend the previous year have re-entered in the current year, while 11,95% of the young women who did attend the previous year have dropped out in the current year.

The control variables, used in all models, are age dummies, year dummies, urban and peri-urban residence dummies, distance to primary road and distance to secondary road. Table 1 presents descriptive statistics of the variables. The most striking observation is perhaps that school attendance is strongly associated with a reduced risk of HIV infection in the raw data. School attendance is, however, likely to decrease with age, so part of the association is clearly due to the fact that the probability of infection increases with age.

**Table 1:** Descriptive statistics.

	Total	Seroconverted	Risk ratio	95% CI of risk ratio
Attend school, n (%)	5,697 (77,59)	96 (1.69)	0.280	[0.213, 0.369]
Do not attend school, n (%)	1,645 (22.41)	99 (6.02)	Comparison group	
Urban, n (%)	209 (2.85)	5 (2.39)	1.798	[0.753, 4.293]
Peri-urban, n (%)	2,296 (31.27)	81 (3.53)	2.651	[2.103, 3.341]
Rural, n (%)	4,837 (65.88)	109 (2.25)	Comparison group	
Distance to primary road, mean (SD)	7.271 (6.734)		0.975	[0.954, 0.997]
Distance to secondary road, mean (SD)	1.453 (1.241)		0.979	[0.872, 1.097]
Age 15, n (%)	572 (7.79)	0 (0.00)	0.000	Not defined
Age 16, n (%)	1,454 (19.80)	8 (0.55)	Comparison group	
Age 17, n (%)	1,358 (18.50)	12 (0.88)	1.606	[0.659, 3.917]
Age 18, n (%)	1,079 (14.70)	23 (2.13)	3.874	[1.740, 8.628]
Age 19, n (%)	836 (11.39)	23 (2.75)	5.000	[2.247, 11.128]
Age 20, n (%)	625 (5.51)	31 (4.96)	9.015	[4.167, 19.500]
Age 21, n (%)	466 (6.35)	23 (4.94)	8.970	[4.040, 19.918]
Age 22, n (%)	366 (4.99)	24 (6.56)	11.918	[5.399, 26.310]
Age 23, n (%)	309 (4.21)	30 (9.71)	17.646	[8.169, 38.117]
Age 24, n (%)	277 (3.77)	21 (7.58)	13.779	[6.166, 30.792]
Year 2005, n (%)	674 (9.18)	7 (1.04)	Comparison group	
Year 2006, n (%)	889 (12.11)	22 (2.47)	2.387	[1.026, 5.553]
Year 2007, n (%)	881 (12.00)	23 (2.61)	2.514	[1.085, 5.823]
Year 2008, n (%)	1,051 (14.31)	20 (1.90)	1.832	[0.779, 4.310]
Year 2009, n (%)	759 (10.34)	23 (3.03)	2.918	[1.260, 6.756]
Year 2010, n (%)	982 (13.38)	25 (2.55)	2.451	[1.066, 5.635]
Year 2011, n (%)	902 (12.29)	29 (3.22)	3.096	[1.364, 7.024]
Year 2012, n (%)	1,204 (16.40)	46 (3.82)	3.679	[1.670, 8.102]
Total sample, n (%)	7,342 (100.00)	195 (2.66)		

The observations are from 2,976 women. The risk ratios were computed using Stata’s glm command with the binomial log link.

## The empirical strategy

We estimate the probability of leaving a state, HIV negative in this instance, at time *t* conditional on not having done so before, Pr[*t*≤*T*<*t*+Δ*t*|*T*≥*t*]. This is the discrete time hazard function. The discrete, rather than continuous, time hazard function is estimated since data was collected at discrete points in time, and, importantly, since the discrete time hazard function can be estimated with any binary variable technique (since it is a conditional probability). When estimating a discrete time hazard, it is essential to control for duration, i.e., how long young women have been at risk of HIV acquisition, as flexibly as possible. This is done with age dummies. The probability to turn HIV positive is estimated using various empirical strategies to evaluate whether there is a causal effect or not. Both HIV incidence and school attendance are binary variables. In our main analysis we therefore estimate the bivariate probit model (BPM), which can handle an endogenous regressor either by the use of exclusion restrictions [18, 19, 20] or by evaluating robustness to selection [17]. Let *y*_*it*_ be HIV incidence for individual *i* in year *t* (note that *i* was uninfected in *t-1*). *S*_*it*_ is school attendance, *x*_*it*_ is a vector of control variables, and *z*_*it*_ is a vector of variables that predict school attendance but not HIV incidence conditional on *s*_*it*_ and *x*_*it*_, i.e., the exclusion restrictions. Then,

$$s_{it}=1\left(x_{it}\delta +z_{it}\gamma +u_{it}>0\right)$$ (1)

$$y_{it}=1\left(x_{it}\pi +s_{it}\beta +\varepsilon_{it}>0\right)$$ (2)

$$\left[\begin{array}{c} u\\ \varepsilon \end{array}\right]∼N\left(\left[\begin{array}{c} 0\\ 0 \end{array}\right],\left[\begin{array}{cc} 1 & \rho \\ \rho & 1 \end{array}\right]\right).$$ (3)

The unobserved determinants of school attendance, *u*, and HIV incidence, *ε*, are assumed to have a joint bivariate normal distribution, and *ρ* is the correlation between them. While logistic regression is more popular than probit regression in epidemiology, it is not an option in this application since there is no analogous bivariate logistic distribution. Consequently, there is no corresponding logit model to the BPM that can handle endogenous regressors.

The correlation between unobserved factors in the schooling- and HIV incidence equations, *ρ*, is an estimated parameter. If *ρ* = 0, there is no selection on unobserved variables and the BPM will provide the same information as estimation of Eq (2) with the (univariate) probit model. If *ρ* is significantly different from zero, we can reject the probit model in favor of the BPM. Our other estimate of interest is *β*, since it shows how school attendance effects the risk of HIV infection. If the BPM is a good description of the data generating process this is the causal average treatment effect [18].

The exclusion restrictions are valid if *z* predicts school attendance, (*γ* ≠ 0), but not HIV incidence conditional on *x* and *s*. However, since it is a challenge to find completely credible exclusion restrictions, we first use the approach developed by Altonji et al. [17], which does not depend on exclusion restrictions. Instead the BPM is treated as under-identified, where *ρ* is not estimated but imposed. Hence we estimate,

$$s_{it}=1\left(x_{it}\delta +u_{it}>0\right)$$ (4)

$$y_{it}=1\left(x_{it}\pi +s_{it}\beta +\varepsilon_{it}>0\right)$$ (5)

$$\left[\begin{array}{c} u\\ \varepsilon \end{array}\right]∼N\left(\left[\begin{array}{c} 0\\ 0 \end{array}\right],\left[\begin{array}{cc} 1 & \rho =\varphi \\ \rho =\varphi & 1 \end{array}\right]\right)$$ (6)

under different assumptions about *φ*. This allows us to evaluate how sensitive the effect of school attendance on HIV incidence is to different degrees of selection on unobserved factors (captured by the correlation of the two error terms). The important task is to select sensible values of *φ*, where Altonji et al. suggest using 0 as a lower bound and the variation explained by the selection on observed control variables as an upper bound [17]. More specifically, the upper bound on *ρ* is *Cov*(*xδ*,*xπ*)/*Var*(*xπ*). This would be the value of *ρ* if all variables were observable and the researcher picked control variables randomly; the selection on excluded (unobserved) variables would asymptotically equal the selection on included (observed) variables. In reality, researchers are likely to do better than a random selection of included control variables. But on the other hand, some important control variables might simply not be available. If the estimated school attendance coefficient is negative and statistically significant when *ρ* is within the lower and upper bound, we can conclude that school attendance probably has a causal protective effect on HIV infection.

Next we estimate the BPM using two exclusion restrictions based on distances to secondary schools. Similar instruments have been used extensively; one example is Alsan and Cutler [13] in their study on schooling and HIV in Uganda. Importantly, we also control for key geographic indicators and it is unlikely that families move to be close to secondary schools.

Distance to nearest secondary school varies between 0 and 10 km, with most of the young women living within 2 km of the nearest secondary school. Since secondary schools are relatively close by, students usually live with their family. We use a dummy that indicates closer than 7 km to the nearest secondary school. School attendance drops from 79% to 57% at the 7 km cut-off, while distance has little predictive power within 7 km to a secondary school. A standard, continuous, measure of distance to the nearest secondary school is a weak predictor of school attendance. This exclusion restriction is obviously not ideal, particularly since few young women live 7 km or more from a secondary school. The second exclusion restriction is the difference (in km) between the nearest and second nearest secondary school. It reflects choice; more schools nearby makes it more likely that a young woman finds a school that suits her.

Since both exclusion restrictions are correlated with other distances and with remoteness, we control for distances (in km) to the nearest primary road and secondary road, and include urban and peri-urban dummies. Conditional on these geographic controls, it could be argued that distance to schools should not be systematically related to the risk of HIV incidence through any other channel than school attendance, and thus that our exclusion restrictions are valid.

Finally, to account for time-constant unobserved factors that matter for selection, we estimate the correlated random effects probit model. With this approach, the unobserved effects that matter for selection are allowed to depend on the group means of the covariates, but are assumed to be uncorrelated with covariates conditional on these means [20, 22]. We estimate

$$y_{it}=1\left(x_{it}\pi +s_{it}\beta +{\bar{x}}_{t}\pi_{a}+{\bar{s}}_{t}\beta_{a}+\varepsilon_{it}>0\right),$$ (7)

assuming that *ρ* = 0 conditional on the means, ${\bar{x}}_{i}$ and ${\bar{s}}_{i}$. The main crucial assumption is that unobserved factors that matter for selection are indeed time-constant, and thereby captured by the group means. Many unobserved factors that could be suspected to matter, such as family background, self-control, time preferences, and ability, should be fairly constant over time. But shocks to young women, or their households, might affect both school attendance and sexual behavior. This would bias the correlated random effects coefficient away from zero. However, measurement errors could be more influential since identification relies on deviations from means, which would bias the coefficient towards zero.

To check the robustness of the results, we also estimate linear probability model versions of all our estimations. Apart from estimating Ordinary Least Squares (OLS) models that control for time-constant unobserved individual factors and IV models, the linear framework allows us to use the approach developed by Oster [21] to evaluate robustness to selection on unobserved variables. This approach differs somewhat from the approach of Altonji et al. [17], though it also relies on the assumption that the selection on observed control variables is informative about potential selection on unobserved variables. S2 Appendix provides a short description of the approach and the results of the estimation of the linear models.

The probit regression is the preferred choice in our application. If it is a good approximation of the data-generating process, it is more efficient than a linear model, and predicted probabilities are bounded within the unit range. Most importantly, the linear probability model is biased when many predicted probabilities are outside of the unit range [35], which they tend to be for low-probability events such as HIV incidence. However, since we cannot be certain that the probit model is a good approximation to the data-generating process, it is useful to check robustness to an alternative specification. Some authors have compared the BPM and linear IV models. Though results are inconclusive, the BPM seems to be preferred when average outcomes are close to 0 as in our case [19, 36].

## Results

### The association between school attendance and HIV incidence

Table 2 shows the probit estimation result of the relationship between school attendance and HIV incidence. The table reports marginal effects (or more correctly the partial effect on HIV incidence of a discrete change in School attendance, evaluated at the mean of the other explanatory variables) and the 95% confidence intervals. There is a strong negative association. Young women who attend secondary school are 1.4 percentage points less likely to be infected with HIV. This is a large effect given the average HIV incidence of 2.7%. In particular, it is noteworthy that the association is robust to the inclusion of a full set of age dummies, since several previous studies use a linear age term or a smaller set of age dummies. The complete results of the regression in Table 2 are available in the S3 Appendix. Complete results from the other regressions are available from the authors on request.

**Table 2:** The association between secondary school attendance and HIV incidence (probit marginal effects^a^).

School attendance	−0.014***
	(0.005)
*95% confidence interval*	[-0.023, -0.004]
*Number of observations*	7,342
*Number of young women*	2,976

The model also includes a constant, age and year dummies, peri-urban or urban residence, and distances to the primary road and the secondary road. Standard errors, clustered at the household level, in parentheses.

*** p<0.01.

a) The reported effect is the percentage point impact of school attendance on the probability of HIV infection.

### Evaluating the impact of selection using Altonji et al.’s approach

In this sub-section, we use the approach developed by Altonji et al. [17] to evaluate the sensitivity of the probit marginal effect to potential selection on unobserved factors [17]. Table 3 presents the marginal effects of school attendance on HIV incidence, with *ρ* equal to 0.00, -0.05, -0.10, -0.15, -0.20, -0.25, -0.30, -0.364. Following the suggestion by Altonji et al. [17], the lower bound is set to zero and the upper bound, -0.364, is set to the selection on observed control variables, more specifically *ρ* = *Cov*(*xδ*,*xπ*)/*Var*(*xπ*), where *Cov*(*xδ*,*xπ*) and *Var*(*xπ*) are obtained from estimations of Eqs (4) and (5) in Section 4. The estimated marginal effect of school attendance on HIV incidence does not appear to be robust to selection. It is statistically insignificant when *ρ* = −0.1, and has become positive already when *ρ* = −0.15. Thus, far less correlation is required to remove the significant marginal effect of schooling on HIV incidence than -0.364, the selection on observed variables.

**Table 3:** Robustness of the impact of secondary school attendance on HIV incidence to selection on unobserved factors (bivariate probit model marginal effects).

Assumed *ρ*	0.00	−0.05	−0.1	−0.15	−0.2	−0.25	−0.3	−0.364^a^
*Panel A*: *Full sample*								
Marginal effect	−0.014***	−0.009**	−0.004	0.000	0.005	0.010**	0.016***	0.023^b^
Standard error	(0.005)	(0.005)	(0.005)	(0.005)	(0.005)	(0.005)	(0.005)	(b)
95% CI	[-0.023, -0.004]	[-0.018, -0.000]	[-0.011, 0.002]	[-0.009, 0.009]	[-0.004, 0.014]	[0.001, 0.020]	[0.007, 0.025]	

Based on constrained bivariate probit estimations. Both the school attendance and the HIV incidence equations include a constant, age and year dummies, peri-urban or urban residence, distances to the primary road and the secondary road. Standard errors are computed with the delta method.

** p<0.05

*** p<0.01.

a) −0.364 is the selection on observed variables.

b) The standard error could not be estimated

### Accounting for selection—Bivariate probit model with exclusion restrictions

To account for selection, we estimate HIV incidence and school attendance jointly in a BPM with exclusion restrictions. As Table 4 reports, there is no evidence for a casual effect: the marginal effect of school attendance has switched sign, but it is not statistically significant. It also shows that both exclusion restrictions are strong predictors of school attendance and their marginal effects have the expected signs. Young women living closer than 7 km to a secondary school are 16.2 percentage points more likely to attend school than young women living farther away. Each extra km between the nearest and second nearest school decreases the probability of school attendance by 1.3 percentage points. The correlation between the unexplained variation in school attendance and the unexplained variation in HIV incidence, *ρ*, is estimated to be -0.24, and it is statistically significant (p-value 0.002). Hence, we can reject the probit model in favor of the BPM (if H0: *ρ* = 0 were true, the probit and BPM would have given the same result). The negative *ρ* means that unobserved factors that increase the probability of school attendance decrease the probability of HIV incidence, which is expected. The size of *ρ*, -0.24, can be compared with the correlation that makes the marginal effect of school attendance statistically insignificant using the approach by Altonji et al., *ρ* = -0.10, and with the correlation that makes the marginal effect switch sign, *ρ* = -0.15. Our best estimate of *ρ* is thus much higher than what is needed to remove the effect of school attendance on HIV incidence.

**Table 4:** The impact of secondary school attendance on HIV incidence using exclusion restrictions (bivariate probit model marginal effects).

*HIV incidence equation*			*95% CI*
	School attendance	0.010	[-0.022, 0.043]
		(0.006)	
*School attendance equation*			
	Nearest secondary school <7km away	0.162***	[0.084 0.233]
		(0.038)	
	Distance between the nearest and second nearest secondary school	−0.013**	[-0.022, -0.002]
		(0.005)	
*ρ*		−0.240	
[p-value of test of *ρ* = 0]		[0.002]	
Number of observations		7,341	
Number of young women		2.975	

Both equations also include a constant, age and year dummies, peri-urban or urban residence, and distances to the primary road and the secondary road. Standard errors, clustered at the household level, in parentheses.

** *p*<0.05

*** *p*<0.01.

### Accounting for selection on time-constant unobserved factors

Next we account for selection on time constant unobserved factors using the correlated random effects probit model. The marginal effect of school attendance on HIV incidence is -0.007, half the size of the one estimated with the probit model in Table 1, and statistically insignificant (Table 5).

**Table 5:** The association between secondary school attendance and HIV incidence (correlated random effect probit marginal effects).

School attendance	−0.007
	(0.006)
*95% confidence interval*	[-0.019, 0.005]
*Number of observations*	7,342
*Number of young women*	2,976

The model also includes a constant, age and year dummies, peri-urban or urban residence, distances to the primary road and the secondary road, and individual level means of all explanatory variables. Mean Standard errors, clustered at the household level, in parentheses.

### Robustness tests

In S2 Appendix we report estimations of linear models that evaluate sensitivity to selection, use instruments to find a causal effect, and control for time-constant unobserved factors. When the linear probability model is estimated with OLS, the marginal effect is slightly larger than in the probit model: HIV incidence is 1.8 percentage points lower among young women attending secondary school. However, the estimated marginal effect in the individual fixed effects estimation is very small and statistically insignificant, supporting earlier findings. The linear instrumental variable model is estimated using the limited information maximum likelihood estimator (LIML). The marginal effect of school attendance on HIV incidence is positive, but the confidence intervals are very wide, including extreme, non-sensible, values on both the negative and positive sides. Using Oster’s (2016) sensitivity analysis, the selection of unobserved variables needed to remove a causal effect of school attendance on HIV incidence is only 0.275 of the selection on observed variables (the benchmark is 1).

In S4 Appendix we report estimations were HIV incidence has been coded differently. Among the women who ever tested positive, 40% tested negative in the previous years, while the other women have missing observations between the year in which they last tested negative and the year in which they tested positive. In the main analysis, the HIV incidence variable is coded 1 the first year in which a woman tested positive. Years between the last HIV-negative test and before the first HIV-positive result are coded as missing. In the robustness estimations in S4 Appendix, we first code HIV incidence as missing also the first year in which a woman tested positive if she did not test negative the year before. Then we completely remove women from the sample for whom we cannot be certain about infection year, i.e. we remove also the observations for years that we know they were HIV negative. For all three approaches were we deal with endogeneity, the results are very similar to those obtained in the main analysis: that is, for the estimated marginal effect of school attendance, the estimated correlation between unobserved factors in the BPM model, and the selection on observed variables used as an upper bound in the sensitivity analysis. However, in the first regression, where we do not account for endogeneity, the estimated marginal effect of school attendance on HIV incidence is much smaller and no longer statistically significant when we use the alternative coding.

## Discussion and conclusion

In spite of recent achievements in providing antiretroviral treatment to an increasing number of people, HIV/AIDS remains a serious challenge in heavily affected sub-Saharan African countries. With a growing number of infected on life-long treatment, the costs are escalating, threatening the achievements in South Africa and several other African countries. Consequently, there is an urgent need to increase investments in prevention. According to UNAIDS, among others, keeping girls in school is one of the most powerful structural interventions for HIV risk reduction among young women across Africa [2, 3, 4].

This study utilizes a longitudinal dataset on HIV incidence from rural KwaZulu-Natal in South Africa, an area with high HIV rates, to evaluate the causal effect of school attendance (keeping girls in school) on HIV incidence among young women. We employ a variety of approaches based on: models that evaluate the sensitivity to selection; models that control for constant unobserved individual effects; and models with exclusion restrictions. All have their virtues and vices, but none suggests a causal effect.

To evaluate the sensitivity to omitted variables, we use the approaches developed by Altonji et al. and Oster, which have been employed in many studies during recent years [17, 21]. Both show that the degree of selection on unobserved factors needed to remove a causal effect is much smaller than the selection on observed variables (the benchmark). In fact, it is much lower than our best estimate of the selection on unobserved variables, from the bivariate probit model.

Approaches that control for time constant unobserved factors rely on within-group variation for identification. They could be biased away from zero due to shocks during the study period, such as loss of income, or psychological stress, which would affect both schooling and sexual behavior. However, measurement error could create a bias that goes in the other direction. Nonetheless, in both the correlated random effects probit and the linear models that control for individual unobserved effects, the marginal effect of school attendance on HIV incidence is small and statistically insignificant. This suggests that selection on time-constant unobserved factors is likely to be a key reason for the correlation between school attendance and HIV infection.

The main limitation of approaches that rely on instrumental variables to identify a causal effect is that the exclusion restrictions can often be discussed. We attempt to alleviate the usual concerns about distance-based instruments by including various geographic control variables, but we cannot be certain that these are sufficient. Moreover, although we have two instruments, the fact that few women are affected by the 7 km cut-off is a disadvantage, in particular in the linear model, where we only estimate the effect on the complying subpopulation. The estimated effects should therefore be interpreted with caution. Nonetheless, the fact that they are positive and statistically insignificant in both the bivariate probit and the linear model corroborate the results of the two other approaches.

Summing up, selection, i.e., unobserved factors, seems to be the main reason behind the negative correlation between school attendance and HIV infection. While it is not possible to completely rule out a causal effect, if there is one, it is probably small. We can only speculate about what these unobserved factors might be, but family background, self-control, time preferences and academic ability are possible candidates. Nonetheless, though our study uses data from a specific place, selection is likely to matter in other places too. Thus, our findings have implications for the interpretation of the multitude of studies that only analyse associations between school attendance and HIV; young women who attend school differ from those who drop out. This means that claims about the protective effects of schooling should be interpreted with care.

At first glance, our results might appear to be in conflict with the study that finds a protective effect of schooling in Botswana [15]. However, it analyzes the impact of increased school attainment on the cumulative risk of HIV infection until the age at which their data was collected, while we analyze the effect of current school attendance. It is also possible that the quality of education is better in Botswana than in our study area in KwaZulu-Natal, and that education needs to be good enough to have a protective effect.

Our results might also appear to be at odds with the study that finds that schooling delays sexual debut in Uganda [14]. However, delayed sexual debut is just one out of many proximate determinants: school attendance could, for example, make some girls delay their sexual debut, while others have more premarital casual relationships, as have been suggested by Duflo et al. [32]. There is support for delayed sexual debut as a consequence of school attendance in our data too, but many of the sexually active girls remain in school, while those who become pregnant, and often drop out, are less likely than other sexually active young women to be HIV infected. These results are not reported, but available from the authors.

Again, the specific setting could also matter. Secondary education might have had a more beneficial impact in Uganda due to the simultaneous massive anti-HIV/AIDS campaigns in Uganda. This would also be consistent with Duflo et al. [32] who found positive effects of school attendance in Kenya only when it was combined with an improved HIV/AIDS curriculum. In South Africa, the Life Orientation Program, which includes HIV education, was introduced in the curriculum in 2000, but the quality of implementation has been questioned [37, 38]. Young women in South Africa are also surrounded by other types of information, which likewise shape their beliefs.

In spite of the many studies on education and HIV incidence, those who go beyond associations and attempt to separate causation from selection are surprisingly few. It is therefore a challenge to draw general conclusions without more research. Nevertheless, given current knowledge we should not expect keeping girls in school to automatically reduce HIV infections. More targeted measures might be needed.

## Supporting information

S1 Appendix The relationship between education and HIV incidence: A conceptual framework. (DOCX)

S2 Appendix Complete Table 2. (DOCX)

S3 Appendix Linear models. (DOCX)

S4 Appendix Alternative coding of HIV incidence. (DOCX)
