# Supplementary material for: Education and HIV incidence among young women in KwaZulu-Natal: An association but no evidence of a causal protective effect
Source: PLoS One. 2019 Mar 4;14(3):e0213056. doi: 10.1371/journal.pone.0213056 (PMC6398860; doi:10.1371/journal.pone.0213056)
Supplement: S1 Appendix — (DOCX) [file pone.0213056.s001.docx]

## S1 The relationship between education and HIV incidence: a conceptual framework

Figure S1 depicts our conceptual framework for the relationship between education and HIV incidence. Starting from the right, sexual behavior is the proximate determinant of HIV incidence, so schooling affects HIV incidence through its impact on sexual behavior. There are various sexual behavior determinants of HIV infection; such as the age of sexual debut, frequency and type of intercourse, number of partners, age of partners (older partners are more likely to be infected) and condom use, and it should be noted that schooling might affect these proximate determinants differently.

The potential impact of schooling on sexual behavior is divided into the short-term effect of attending school rather than doing some other activity, and the potentially more long-term effect of human capital accumulation. The short-term school attendance channel may reduce HIV risk because current school attendance influences social and sexual networks (Hargreaves et al., 2007), or because there is an incarceration effect, i.e., school attendance reduces the time available for risky sex (Black et al., 2008; Alsan and Cutler, 2013). Another potentially protective mechanism is HIV prevention campaigns, which often are school-based. However, the impact of school attendance is likely to vary both depending on the school and on the activities young women pursue when they do not attend school; for instance, walking to school might both expose young women to the risk of being abused as well as provide opportunities for transactional sex. Thus, the short-term school attendance effect could go either way.

Since education increases human capital, it raises the opportunity cost of infection and shortened life expectancy, which in turn should lead to less risky sexual behavior (Fortson, 2008; Oster, 2012). Education also makes people more able to adopt protective behaviors, and some studies focus on the ability to process and acquire new information and change behavior in accordance with that new information (De Walque, 2007; Hargreaves et al., 2008). If the protective effect is due to human capital accumulation, we should expect to see most of the positive effects after the women have left school. There could also be indirect effects via the labor or marriage markets in the long run.

Figure S1: The relationship between schooling and HIV incidence

Human

capital

channel

Attendance

channel

Pregnancy

Figure S1 also shows that social background factors and individual endowments matter for both selection into education and for sexual behavior. Young women who come from more favorable social- and family backgrounds are more likely to attend secondary school. And as documented in several studies, their sexual behavior is also often less risky (Eaton et al., 2003). Individual endowments include genetic and early childhood factors that are partly exogenous to schooling, such as time preferences, self-control and academic ability.^[[1]](#footnote-1)^ Girls who value the future more are more likely to attend school and might also be more careful not to contract HIV. Similarly, girls who are better academically, and who therefore attend school, might also be more able to process HIV and family planning information and to change their behavior in accordance with that information. Finally, sexual behavior may affect schooling negatively, particularly through pregnancy, which is an important reason for dropping out of school.

***New references***

Black, Sandra E. and Devereux, Paul J. and Salvanes, Kjell G (2008). Staying in the Classroom and out of the Maternity Ward? The Effect of Compulsory Schooling Laws on Teenage Births, *The Economic Journal* 118(530): 1025-1054.

Eaton, L., Flisher, A. J., & Aarø, L. E. (2003). Unsafe Sexual Behaviour in South African Youth. *Social Science & Medicine*, 56(1), 149-165.

Fortson, J. (2008). The Gradient in Sub-Saharan Africa: Socioeconomic Status and HIV/AIDS. *Demography,* 45(2):303 -322

Hargreaves, J.R., L.A. Morison, J.C. Kim, C. P. Bonell, J. D. H. Porter, C. Watts, J. Busza, G. Phetla, P. M. Pronyk (2008). Evidence-based public health policy and practice: The association between school attendance, HIV infection and sexual behaviour among young people in rural South Africa. *Journal of Epidemiological Community Health,* 62(2): 113-119.

Oster, E. (2012). HIV and sexual behavior change: Why not Africa? *Journal of Health Economics* 31(1): 35-49.

1. Time-preferences, self-control and academic ability might change over time, and could potentially be affected by schooling. Such effects would be part of the human capital channel. However, a large part of these endowments are determined at early age. [↑](#footnote-ref-1)
