# Supplementary material for: Education and HIV incidence among young women in KwaZulu-Natal: An association but no evidence of a causal protective effect
Source: PLoS One. 2019 Mar 4;14(3):e0213056. doi: 10.1371/journal.pone.0213056 (PMC6398860; doi:10.1371/journal.pone.0213056)
Supplement: S2 Appendix — (DOCX) [file pone.0213056.s002.docx]

**S2: Complete table 2**

| Table A1: The association between secondary school attendance and HIV incidence (probit marginal effects) | |
| --- | --- |
| School attendance | -0.014*** |
|  | (0.005) |
| Age 16 | 0.183*** |
|  | (0.014) |
| Age17 | 0.192*** |
|  | (0.014) |
| Age 18 | 0.212*** |
|  | (0.014) |
| Age 19 | 0.216*** |
|  | (0.015) |
| Age 20 | 0.230*** |
|  | (0.015) |
| Age 21 | 0.228*** |
|  | (0.015) |
| Age 22 | 0.236*** |
|  | (0.016) |
| Age 23 | 0.245*** |
|  | (0.016) |
| Age 24 | 0.236*** |
|  | (0.016) |
| Year 2006 | 0.021** |
|  | (0.010) |
| Year 2007 | 0.020** |
|  | (0.010) |
| Year 2008 | 0.014 |
|  | (0.010) |
| Year 2009 | 0.020* |
|  | (0.010) |
| Year 2010 | 0.022** |
|  | (0.010) |
| Year 2011 | 0.029*** |
|  | (0.010) |
| Year 2012 | 0.033*** |
|  | (0.010) |
| Peri-urban | 0.010* |
|  | (0.005) |
| Urban | 0.002 |
|  | (0.013) |
| Distance to the primary road | -0.000 |
|  | (0.000) |
| Distance to the secondary road | -0.000 |
|  | (0.002) |
| *N* | 7,342 |
| Standard errors, clustered at the household level, in parenthesis. * *p*<0.1; ** *p*<0.05; *** *p*<0.01 | |
