# Supplementary material for: Education and HIV incidence among young women in KwaZulu-Natal: An association but no evidence of a causal protective effect
Source: PLoS One. 2019 Mar 4;14(3):e0213056. doi: 10.1371/journal.pone.0213056 (PMC6398860; doi:10.1371/journal.pone.0213056)
Supplement: S3 Appendix — (DOCX) [file pone.0213056.s003.docx]

**S3: Linear models**

In this section, we use LPMs to analyze the data (Table A3). When the LPM is estimated with OLS, the marginal effect is slightly larger than in the probit model: HIV incidence is 1.8 percentage points lower among young women attending school.

Next we estimate a model with individuals fixed effects. If characteristics that determine both school dropout and HIV infection are constant over time, this gives us a casual effect. However, even if selection is not completely eliminated, fixed effects estimation should be an improvement over the OLS estimation and the estimated coefficients should be closer to the casual ones.

The estimated marginal effect in the individual fixed effects estimation is very small and statistically insignificant.

| Table A2: Linear estimations of the impact of secondary school attendance on HIV incidence | | | |  |
| --- | --- | --- | --- | --- |
|  | OLS | Individual fixed effects | IV - LIML | |
| School attendance | −0.018** | −0.001 | 0.005 | |
|  | (0.007) | (0.009) | (0.076) | |
| *R*^2^ | 0.03 | 0.076 |  | |
| *Number of observations* | 7,342 | 7,342 | 7,342 | |
| *Number of women* | 2,976 | 2,976 | 2,976 | |
| Note: All models also include a constant, age and year dummies, peri-urban or urban residence, distances to the primary road and the secondary road. Standard errors, clustered at the individual level, in parentheses. * *p*<0.1; ** *p*<0.05; *** *p*<0.01. The instruments are nearest secondary school <7km away, and distance between the nearest and second nearest secondary schools. | | | |  |

The model with instrumental variables is estimated with the limited information maximum likelihood estimator (LIML). The first stage regression results (Table A4) show that the instrument coefficients are statistically significant and of the expected signs. Our F-statistic is 10.20. This should be compared to the reported weak instrument critical values developed by Stock and Yogo (2005).^[[1]](#footnote-1)^ The critical values depend on both the specific IV estimator and the number of instruments used. With LIML and two instruments, the critical value for 10% maximal IV size is 8.68, and the one for a 15% maximal IV bsize is 5.33. Hence, our instruments are strong enough.

| Table A3: First stage regression results | |
| --- | --- |
| Nearest school closer than 7 km | 0.196*** |
|  | (0.044) |
| Distance between the nearest and second nearest secondary school | -0.015*** |
|  | (0.005) |
| *R*^2^ | 0.28 |
| *N* | 7,341 |
| F – test of excluded instruments | 10.20 |
| Stock-Yogo weak ID test critical value for 10% maximal IV size | 8.68 |
| Stock-Yogo weak ID test critical value for 15% maximal IV size | 5.33 |
| The model also includes a constant, age and year dummies, peri-urban or urban residence, distances to the primary road and the secondary road. Standard errors, clustered at the household level, in parenthesis. * *p*<0.1; ** *p*<0.05; *** *p*<0.01 | |

The marginal effect of school attendance on HIV incidence is positive (Table 3). However, the confidence intervals are very wide, including extreme, non-sensible, values on both the negative and positive sides. This is in line with Chiburis et al. (2012), who show that LPIVM estimations sometimes result in confidence intervals that are too broad for meaningful hypothesis testing.

Finally, we use the approach developed by Oster (2016) to evaluate robustness to selection on unobserved factors. This approach differs somewhat from the Altonji et al. (2005) approach, but it also relies on the assumption that the selection on observed control variables is informative about potential selection on unobserved variables.^[[2]](#footnote-2)^ Specifically, the selection on unobserved variables is assumed to be proportional to the selection on observed variables, where δ is the coefficient of proportionality. An assumption is also needed about the R-squared that would be obtained if all relevant variables, both observed and unobserved, were included in the model, denoted maximum R-squared. The main challenges are therefore to decide on the coefficient of proportionality and maximum R-squared. We follow Altonji et al. (2005) and Oster (2016) in assuming that the selection on unobserved variables is at most as large as the selection on observed variables, i.e., δ=1. The maximum R-squared is likely to be much lower than 1 in our case, both because our dependent variable is binary and because there is a great deal of idiosyncratic variation in HIV incidence. We follow Oster (2016) and set the maximum R-squared to the R-squared obtained with the fixed effects estimator, which is 0.070.

Oster’s approach is used in two ways. First, we compute bounds on the coefficient, given the coefficient of proportionality $\delta.$ We get the lower bound (our effect is negative) by assuming no selection on unobserved factors, i.e., $\delta=0,$ which is the OLS coefficient. To get the upper bound, we assume $\delta=1.$ The second approach is to compute the value of $\delta$ that makes the estimated coefficient equal to zero. This tells us how much selection on unobserved factors, in comparison to the selection on observed control variables, is needed to explain away the estimated impact of school attendance on HIV incidence. Outcomes of both approaches are reported in Table A5. The upper and the lower bounds are 0.063 and -0.018. The marginal effect of school attendance switches sign when δ=0.275. Because the upper bound is large and relatively little selection bias is needed to remove the school attendance effect there is no support for a causal effect of schooling on HIV.

| Table A4: Robustness of the impact of secondary school attendance on HIV incidence to selection on unobserved factors | |
| --- | --- |
| Assumed maximum *R*^2^ | 0.076 |
| Uncontrolled *R*^2^ | 0.013 |
| Controlled *R*^2^ | 0.031 |
| *Coefficient bounds* |  |
| Upper bound (δ=1) | 0.063 |
| Lower bound (δ=0) | −0.018 |
| *How much selection to explain away the estimated OLS effect* |  |
| δ making the estimated effect = 0 | 0.275 |

***New reference***

Stock, J. H., and M. Yogo (2005). Testing for weak instruments in linear IV regression. *Identification and inference for econometric models: Essays in honor of Thomas Rothenberg*. Cambridge University Press.

1. The IV estimator will always contain a bias if the endogenous regressor is indeed endogenous. The size of this bias depends on the strength of the instrument. One definition of a strong instrument is that the actual size of a Wald test is close to the conventional. The maximum IV estimator size is expressed in terms of the maximum actual size of a 5% level Wald test. [↑](#footnote-ref-1)
2. This does not imply correlation between observed controls and unobserved factors; in fact, if unobserved factors were highly correlated with the observed control variables, we would not have a selection problem. We therefore only need to consider the orthogonal parts of the unobserved factors and the direction of their impact relative to the observed (control) variables. [↑](#footnote-ref-2)
