# Supplementary material for: Education and HIV incidence among young women in KwaZulu-Natal: An association but no evidence of a causal protective effect
Source: PLoS One. 2019 Mar 4;14(3):e0213056. doi: 10.1371/journal.pone.0213056 (PMC6398860; doi:10.1371/journal.pone.0213056)
Supplement: S4 Appendix — (DOCX) [file pone.0213056.s004.docx]

## S4: Alternative coding of HIV incidence

Tables A5 to A8 show the estimations on a sample with an alternative coding of the HIV incidence variables. HIV incidence is 0 if a woman tested negative in that year or in a later year. It is 1 if she first tested positive in that year and tested negative in the previous year.

| Table A5: The association between secondary school attendance and HIV incidence (probit marginal effects) | |
| --- | --- |
| School attendance | −0.004 |
|  | (0.003) |
| *Number of observations* | 7,228 |
| *Number of young women* | 2,944 |
| Note: The model also includes a constant, age and year dummies, peri-urban or urban residence, and distances to the primary road and the secondary road. Standard errors, clustered at the household level, in parentheses. * *p*<0.1; ** *p*<0.05; *** *p*<0.01. | |

| Table A6: Robustness of the impact of secondary school attendance on HIV incidence to selection on unobserved factors (bivariate probit model marginal effects) | | | | | | | | | |
| --- | --- | --- | --- | --- | --- | --- | --- | --- | --- |
| Assumed *ρ* | | 0.00 | −0.05 | −0.1 | −0.15 | −0.2 | −0.25 | −0.3 | −0.348^a^ |
| *Panel A: Full sample* | | |  |  |  |  |  |  |  |
| Marginal effect | −0.004 | | −0.001 | 0.001 | 0.003 | 0.006 | 0.008** | 0.011^b^ | 0.015** |
| Standard error | (0.003) | | (0.003) | (0.003) | (0.003) | (0.003) | (0.004) | $\cdot$ | (0.008) |
| Note: Based on constrained bivariate probit estimations. Both the school attendance and the HIV incidence equations include a constant, age and year dummies, peri-urban or urban residence, distances to the primary road and the secondary road. Standard errors are computed with the delta method.  a) −0.348 is the selection on observed variables.  b) The standard error could not be estimated | | | | | | | | | |

| Table A7: The impact of secondary school attendance on HIV incidence using exclusion restrictions (bivariate probit model marginal effects) | | |
| --- | --- | --- |
| *HIV incidence equation* |  |  |
|  | School attendance | 0.012 |
|  |  | (0.008) |
| *School attendance equation* |  |  |
|  | Nearest secondary school <7km away | 0.160*** |
|  |  | (0.038) |
|  | Distance between the nearest and second nearest secondary school | −0.013** |
|  |  | (0.005) |
| *ρ* |  | −0.306 |
| [p-value of test of *ρ*=0] |  | [0.005] |
| Number of observations |  | 7,227 |
| Number of young women |  | 2,943 |
| Note: Both equations also include a constant, age and year dummies, peri-urban or urban residence, and distances to the primary road and the secondary road. Standard errors, clustered at the household level, in parentheses. * *p*<0.1; ** *p*<0.05; *** *p*<0.01. | | |

| Table A8: The association between secondary school attendance and HIV incidence (correlated random effect probit marginal effects) | |
| --- | --- |
| School attendance | 0.000 |
|  | (0.004) |
| *Number of observations* | 7,228 |
| *Number of young women* | 2,944 |
| Note: The model also includes a constant, age and year dummies, peri-urban or urban residence, distances to the primary road and the secondary road, and individual level means of all explanatory variables. Mean Standard errors, clustered at the household level, in parentheses. * *p*<0.1; ** *p*<0.05; *** *p*<0.01. | |

Tables A9 to A12 show the estimations on a sample with yet another alternative coding of the HIV incidence variables. Women with missing information between the last year they tested negative and the first year they tested positive are removed from the estimation sample.

| Table A9: The association between secondary school attendance and HIV incidence (probit marginal effects) | |
| --- | --- |
| School attendance | −0.004 |
|  | (0.003) |
| *Number of observations* | 6,737 |
| *Number of young women* | 2,709 |
| Note: The model also includes a constant, age and year dummies, peri-urban or urban residence, and distances to the primary road and the secondary road. Standard errors, clustered at the household level, in parentheses. * *p*<0.1; ** *p*<0.05; *** *p*<0.01. | |

| Table A10: Robustness of the impact of secondary school attendance on HIV incidence to selection on unobserved factors (bivariate probit model marginal effects) | | | | | | | | | |
| --- | --- | --- | --- | --- | --- | --- | --- | --- | --- |
| Assumed *ρ* | | 0.00 | −0.05 | −0.1 | −0.15 | −0.2 | −0.25 | −0.3 | −0.347^a^ |
| *Panel A: Full sample* | | |  |  |  |  |  |  |  |
| Marginal effect | −0.004 | | −0.002 | −0.001 | 0.003 | 0.006 | 0.009** | 0.012^b^ | 0.015*** |
| Standard error | (0.003) | | (0.003) | (0.003) | (0.003) | (0.004) | (0.004) | $\cdot$ | (0.002) |
| Note: Based on constrained bivariate probit estimations. Both the school attendance and the HIV incidence equations include a constant, age and year dummies, peri-urban or urban residence, distances to the primary road and the secondary road. Standard errors are computed with the delta method.  a) −0.347 is the selection on observed variables.  b) The standard error could not be estimated | | | | | | | | | |

| Table A11: The impact of secondary school attendance on HIV incidence using exclusion restrictions (bivariate probit model marginal effects) | | |
| --- | --- | --- |
| *HIV incidence equation* |  |  |
|  | School attendance | 0.012 |
|  |  | (0.008) |
| *School attendance equation* |  |  |
|  | Nearest secondary school <7km away | 0.152*** |
|  |  | (0.038) |
|  | Distance between the nearest and second nearest secondary school | −0.011** |
|  |  | (0.005) |
| *ρ* |  | −0.308 |
| [p-value of test of *ρ*=0] |  | [0.004] |
| Number of observations |  | 6,736 |
| Number of young women |  | 2,708 |
| Note: Both equations also include a constant, age and year dummies, peri-urban or urban residence, and distances to the primary road and the secondary road. Standard errors, clustered at the household level, in parentheses. * *p*<0.1; ** *p*<0.05; *** *p*<0.01. | | |

| Table A12: The association between secondary school attendance and HIV incidence (correlated random effect probit marginal effects) | |
| --- | --- |
| School attendance | 0.001 |
|  | (0.007) |
| *Number of observations* | 6,737 |
| *Number of young women* | 2,709 |
| Note: The model also includes a constant, age and year dummies, peri-urban or urban residence, distances to the primary road and the secondary road, and individual level means of all explanatory variables. Mean Standard errors, clustered at the household level, in parentheses. * *p*<0.1; ** *p*<0.05; *** *p*<0.01. | |
